# Factors associated with help-seeking by women facing intimate partner violence in India: findings from National Family Health Survey-5 (2019–2021)

**DOI:** 10.1186/s44263-024-00056-3

**Published:** 2024-04-17

**Authors:** Rakhi Ghoshal, Priti Patil, Isha Sinha, Anita Gadgil, Priyansh Nathani, Nethra Jain, Premkumar Ramasubramani, Nobhojit Roy

**Affiliations:** 1 Gender Justice, CARE, Kolkata, 700039 India; 2 Department of Statistics, BARC Hospital, Mumbai, 400094 India; 3 State Resource Centre, Nalanda Medical College and Hospital, Patna, 800007 India; 4 The George Institute of Global Health, New Delhi, 110025 India; 5 Hinduhridaysamrat Balasaheb Thackeray Medical College and Dr R N Cooper Municipal General Hospital, Mumbai, 400056 India; 6 Grant Government Medical College and Sir J.J. Group of Hospitals, Mumbai, 400008 India; 7 Department of Community Medicine, Mahatma Gandhi Medical College and Research Institute, Puducherry, 607402 India; 8 Department of Global Public Health, Karolinska Institutet, 17177 Solna, Sweden

**Keywords:** Domestic violence, Family violence, Empowerment, Alcohol consumption

## Abstract

**Background:**

Intimate partner violence (IPV) against women has harmful effects on their psychological and physical health. However, help-seeking for IPV is significantly low among women in the Indian context. This study examines the different factors that influence help-seeking behaviour among women in India. It also studies associations of the type of IPV with the source of help.

**Methods:**

The study analyses data from the fifth round of the National Family Health Survey that was conducted in India (2019–2021). Independent variables were categorized at individual, relationship-household and community levels. The Stata 14.2 software was used to calculate the prevalence ratios and their corresponding 95% confidence intervals. Variables with *p*-values less than 0.05 were considered statistically significant. Poisson regression helped identify factors associated with help-seeking.

**Results:**

Results indicate that among 72,320 women aged 18–49 years, 17,765 women ever-faced IPV. Of them, 14.2% of women who faced either physical and/or sexual IPV sought any help. Husband’s consumption of alcohol almost doubled the likelihood of help-seeking among women (19.91%), compared to women whose husbands did not drink alcohol (10.19%). Witnessing parental IPV also increased the odds (17.26%) of help-seeking. Women who were not empowered were more likely to seek help (14.11%) compared to women who were empowered (12.56%). The police were the predominant source where women went for formal help (6.94 to 8.43%), followed by doctors (1.84 to 2.71%). Close to 1 in 4 women (22.5%) sought help for sexual IPV, while 14.4% of women sought help for physical IPV. Around 95% of all women who faced IPV sought informal help, with 3 in 5 of them approaching their own families, and 3 in 10 approaching their in-laws or marital families.

**Conclusions:**

Two significant factors that associate positively with help-seeking by women facing IPV are husbands’ alcohol consumption and witnessing parental IPV. Most women preferred informal help from the natal family, while among formal providers, the police were the foremost choice. Programmes and initiatives to build capacities of communities, and of police to respond to women seeking help for IPV, would enable more women to reach out for help.

**Supplementary Information:**

The online version contains supplementary material available at 10.1186/s44263-024-00056-3.

## Background

Intimate partner violence (IPV) against women is a global public health problem that adversely affects a woman’s psychological and physical health [1–3]. Often, the health impacts are intergenerational, negatively affecting the mental and developmental health of children growing up in such households [4, 5]. Globally, 35% of women face IPV at least once in their lifetimes [6], though at 42%, the burden is significantly higher in South Asia [7]. To emphasise the burden of violence against women, including IPV, the United Nations Entity for Gender Equality and the Empowerment of Women (UN Women) has termed it a ‘shadow pandemic’ [8]—the adjective ‘shadow’ foregrounding the silent and socially accepted nature of this form of violence against women.

However, globally, the proportion of women seeking help for IPV is not commensurate with the high burden of IPV. According to a World Health Organization (WHO) study, up to 95% of survivors of IPV in developing countries never seek help from any source [9]. Sources of help can be formal and institutional, such as the police, legal support, healthcare providers or informal such as friends, neighbours and family members [9]. The WHO study [9] finds that among the roughly 5% of women who do seek help, very few seek formal help [2, 4, 10–13].

**Fig. 1:**
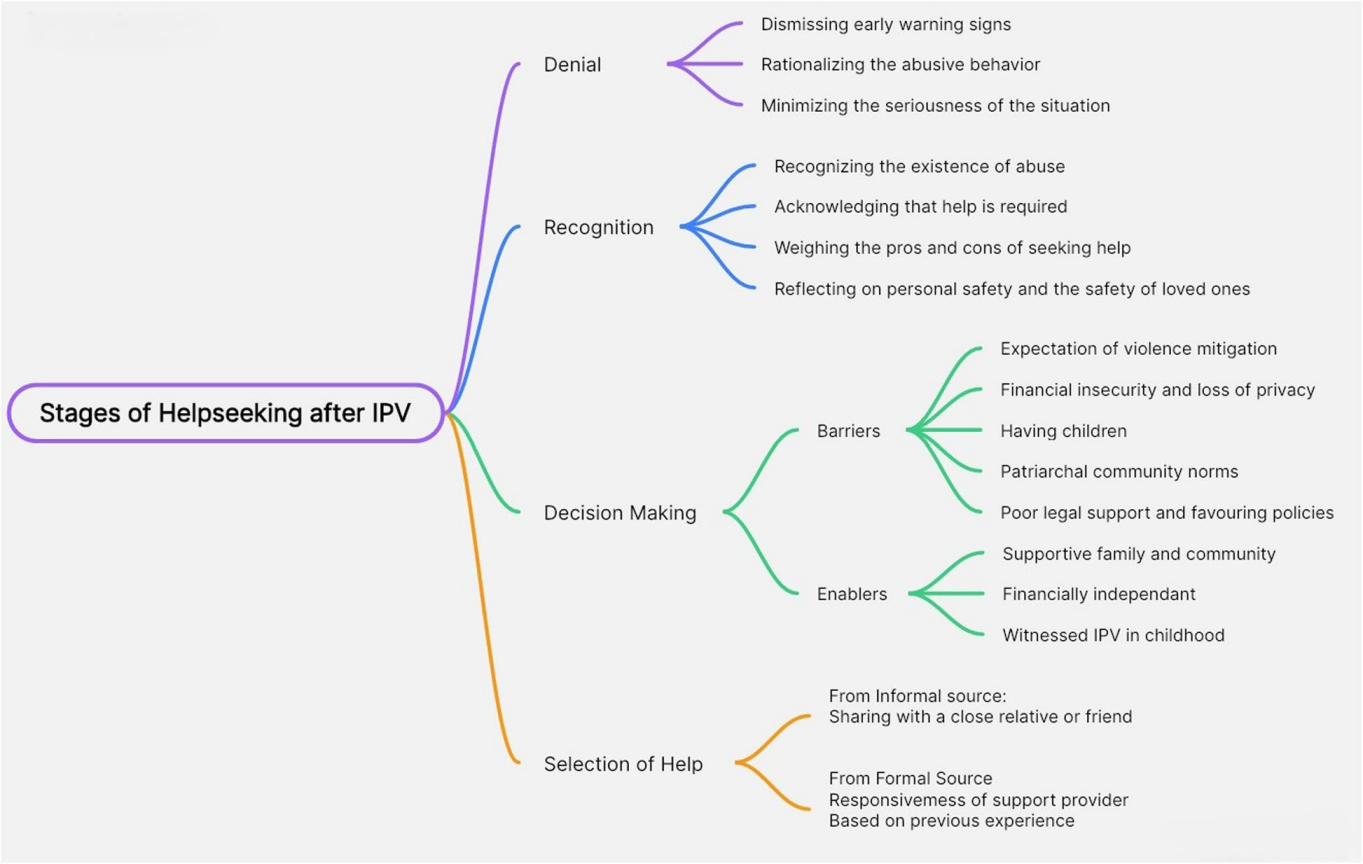
Pathways for help-seeking. Source: prepared by co-authors after synthesizing information and evidence from references [4, 5, 9, 10, 12, 17, 18, 22, 23, 27, 28]

Help-seeking for IPV is a three-stage process [14]. The first stage involves recognizing and defining the abuse. In societies where IPV is normalized, recognition is often delayed or denied [15–17]. The second stage involves making the decision to seek help, which is influenced by various factors [18]. At the individual level, fear of privacy loss, health impacts and past exposure to parental IPV can affect this decision-making [4, 5, 15, 19, 20]. Relationship and household level factors, including the husband’s occupation and frequency of abuse can play a decisive role; the presence of children is known to both drive the woman to seek help and defer help-seeking [16, 20–24]. Community-level factors, such as community support and the presence of local organizations supporting women facing IPV, can positively impact the decision-making process [10, 12, 25–28]. The final stage of help-seeking is about selecting a source of help, and this selection can be influenced by the severity of abuse faced, anticipated response of family members after disclosing the IPV, the woman’s awareness about existing sources and her access to them and her past help-seeking experiences if any [4, 9, 12, 16, 24]. Figure 1 schematically represents the three stages of help-seeking and presents some of the barriers and enablers influencing each stage.

A study from Pakistan [29] found that only 2% of women who faced IPV ever sought formal help. A study [12] analysing help-seeking behaviour across 31 developing countries found that informal help-seeking was highest at 61% in Sierra Leone and lowest at less than 18% in Mali and Tajikistan; overall, formal help-seeking was less than 10%. Additional evidence from the developing world such as from Latin America [30], Uganda [31], Nigeria [16], Ghana [22], and specifically from South Asian countries such as Bangladesh [2, 32], Pakistan [33] and India [4, 10, 11, 15, 34] identifies similar trends, viz. overall low rates of help-seeking and lower rates of formal help-seeking. These studies have found some of the predominant factors for low help-seeking to include non-recognition of the abuse, fear of retaliation by the abuser, anticipated social stigma after disclosure, lack of support by the family, lack of alternative household or economic resources, concern about the future of children and perceived apathy of the formal system. Many of these studies also found that women who were more empowered were less likely to seek help [2, 11, 34, 35]. Studies specifically from India [4, 10, 11, 15] found that between 14 and 24% of women sought any form of help, and less than 2% sought formal help. Between 2005–2006 and 2015–2016, the rate of formal help-seeking in India dropped from 2 to less than 1% [10]. Against this drop, it becomes important to examine the rates of help-seeking in the present time.

The present study aims to examine the rates of help-seeking by women facing IPV in India. Using the most recent population-level dataset (2019–2021) [36], the study analyses the factors that influence help-seeking and examines the associations of the type of IPV, i.e. physical and/or sexual IPV, with the source of help, i.e. formal and/or informal help.

## Methods

### Setting and design

This study used data from the fifth round of the National Family Health Survey (NFHS), 2019–2021 [37]. The NFHS is a large-scale, population-based demographic health survey conducted in India to collect individual-level data at national, state and district levels. Modelled on the Demographic Health Survey (DHS), the NFHS is carried out under the aegis of the Ministry of Health and Family Welfare, India, and is implemented by the International Institute for Population Sciences (IIPS), Mumbai, India. The NFHS provides comprehensive information on population, health and nutrition indicators, which are essential for understanding the health and well-being of the population. The survey covers a wide range of topics, including fertility, family planning, maternal and child health, nutrition, immunization, HIV/AIDS awareness and domestic violence. The fifth round of NFHS (2019–2021) presents data from 28 states and 8 Union Territories in India.

Domestic violence questions were included at the state level (in the state module). A random subsample of 15% of households, drawn from the overall sample, was selected for the implementation of the state module. Weights were applied for the selection of only one woman per household and to guarantee that the domestic violence subsample accurately represented the national population. Weights are calculated based on the probability of selection at each stage of sampling, adjusted for non-response and post-stratified to match known population distributions.

The National Family Health Survey (NFHS) follows global standards for ensuring the privacy of data and confidentiality of survey respondents, in alignment with global guidelines [38] for conducting surveys with women facing IPV. Accordingly, the NFHS demarcates only one eligible woman per household for an interview for domestic violence.

### Participants

**Fig. 2:**
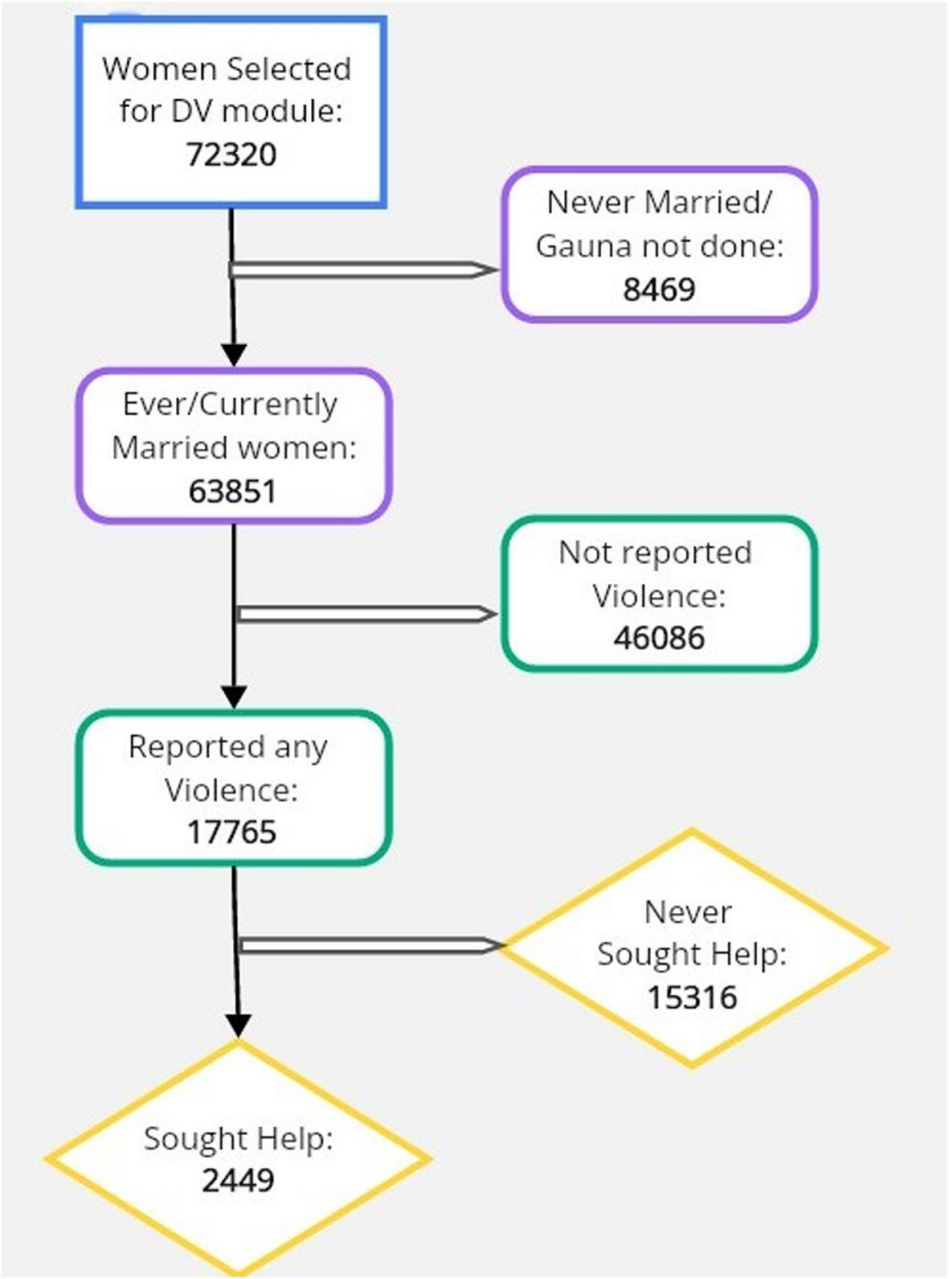
Participant selection

In this present round of the NFHS, 636,699 households including 724,115 women were surveyed. Questions on domestic violence (IPV) were administered to 72,320 women (15% sub-sample) in the 18–49 age group. Of them, 63,851 (88.28%) reported as currently married or ever-married. Among them, 17,765 faced any IPV, and 2449 sought help (Fig. 2).

### Independent variables

The study categorized independent variables at individual, relationship, household and community levels [18]. Individual level variables included the age of woman, marital status, highest level of education and employment status. Relationship and household-level variables included the husband’s highest level of education, comparative earnings of woman and husband, number of living children, woman’s ownership of land or house, husband drinking alcohol, women empowerment (if she made decisions about her own health, large purchases and her mobility), justification of wife-beating/IPV, wealth quintile and experience of witnessing parental IPV. Community-level variables included type of residence.

### Outcome variables

Questions on help-seeking [39] were asked only to women who responded with at least one ‘yes’ to questions on experiencing physical or sexual IPV. Accordingly, outcome variables for this study included help-seeking for physical and/or sexual IPV. Sources of help included family members or neighbours, social service organizations or formal systems such as police or medical personnel, among others. The categorization of sources as either formal or informal was done to sync with existing literature on help-seeking for IPV [4, 11, 14, 35, 40].

### Analysis

The study uses the Stata 14.2 (StataCorp, College Station, TX, USA) software for statistical analysis. The dataset was declared as survey data by adjusting for the sample weights and clusters in the sample design using the ‘svyset’ command. The descriptives were summarized as mean with standard deviation (SD) for continuous variables and frequencies with proportions for categorical variables. The prevalence of help-seeking among women who had experienced physical and/or sexual IPV was reported with 95% confidence interval (CI). Poisson regression was performed to determine the factors associated with the help-seeking.

Poisson regression was applied instead of logistic regression as the former provides prevalence ratio (PR) and the latter provides odds ratio (OR). The NFHS is a cross-sectional survey which provides the prevalence of outcomes, specifically the discrete number of cases where the woman faced either physical or sexual IPV and sought help from at least one source within a specified period. This aligns well with the assumptions of the Poisson regression model. Poisson regression provides a direct way of estimating adjusted prevalence ratio which is a key parameter of interest in this study. Variables with *p*-values less than 0.20 in the univariate model were considered in the multivariate regression model. The association was reported in terms of unadjusted and adjusted PR with 95% CI. Variables with *p*-values less than 0.05 were considered statistically significant factors associated with the outcome.

## Results

The study found that of all women who faced physical and/or sexual IPV (*N* = 17,765), 14.2% (2449) sought help, implying that 85.8% of women did not seek help.

### Demographic characteristics of the study population and prevalence of help-seeking

At the individual level, women who were formerly married had higher odds of help-seeking (25.24%) compared to those who were still married (13.10%). Women with higher secondary level of education (16.73%) and those currently working (18.90%) were more likely to seek help compared to their counterparts.

At the relationship-household level, the likelihood of help-seeking was higher among women who earned more than their husbands (23.22%). Conversely, the rate of help-seeking decreased for women with more number of children: 18.07% of women with no children sought help while only 12.41% with more than two children did. Women whose husbands drank alcohol were twice as likely (19.91%) to seek help compared to those whose husbands did not drink (10.19%). Women who were not empowered were more likely to seek help (14.11%) compared to women who were (12.56%). Women from the richest wealth quintile sought the most help (19.14%), and those who witnessed parental IPV were more likely to seek help (17.26%) than those who did not witness parental IPV (12.54%).

At the community level, a distinct urban–rural disparity in help-seeking behaviour was observed. Urban women showed a higher likelihood of seeking help (17.5%) compared to their rural counterparts (1%). Table 1 presents the demographic characteristics of all women who faced IPV and the prevalence of help-seeking. For the full table see Additional file 1: Table S1.

**Table 1:** Demographic characteristics of all women who faced IPV (*N* = 17,765) and prevalence of help-seeking

Description	All women who faced physical and/or sexual violence		Women who sought help	
	***n***	**% (95% CI)**	***n***	**% (95% CI)**
Overall	17765		2449	14.2
**Individual level**				
Marital status of women				
Currently married	16502	91 (90.6–91.4)	2129	13.1 (11.8–14.4)
Formerly married	1263	9 (8.6–9.4)	320	25.2 (23.6–26.9)
Highest educational level of women				
No education	6671	37.3 (36.6–38.0)	904	13.6 (12.3–14.9)
Primary	2911	15.8 (15.2–16.3)	411	13.6 (12.2–14.9)
Secondary	7149	41.4 (40.6–42.1)	994	14.7 (13.3–16.0)
Higher	1034	5.6 (5.3–6.0)	140	16.7 (15.3–18.2)
Currently working				
No	11,272	63.5 (62.8–64.2)	1311	11.5 (10.3–12.7)
Yes	6493	36.5 (35.8–37.2)	1138	18.9 (17.4–20.4)
**Relationship-household level**				
Earns more than husband				
More than him	1131	6.4 (6.0–6.7)	215	23.2 (21.6–24.8)
Less than him	3260	17.9 (17.4–18.5)	553	17.1 (15.6–18.5)
About the same	973	5.5 (5.1–5.8)	123	14.3 (13.0–15.7)
Husband does not bring in money	322	1.7 (1.5–1.9)	61	16.1 (14.7–17.5)
Do not know	66	0.3 (0.2–0.4)	10	39.3 (37.5–41.2)
Number of living children				
0	1063	6.1 (5.7–6.4)	190	18.1 (16.6–19.6)
≤ 2	9326	52.1 (51.4–52.8)	1279	15.2 (13.8–16.6)
> 2	7376	41.8 (41.1–42.5)	980	12.4 (11.1–13.7)
Husband drinks alcohol				
No	9838	58.8 (58.0–59.5)	959	10.2 (9.0–11.4)
Yes	7927	41.3 (40.5–42.0)	1490	19.9 (18.4–21.5)
Women empowerment (decision about own health, large purchase, visit to family)				
No	5619	32.1 (31.4–32.7)	812	14.1 (12.8–15.5)
Yes	10883	58.9 (58.2–59.6)	1317	12.6 (11.3–13.8)
Wealth quintile				
Poorest	5029	25.5 (24.9–26.2)	636	13.5 (12.1–14.8)
Poorer	4565	25.6 (25.0–26.2)	611	12.3 (11.0–13.6)
Middle	3723	21.7 (21.1–22.2)	525	15.2 (13.8–16.5)
Richer	2828	17.1 (16.6–17.7)	401	14.0 (12.7–15.4)
Richest	1620	10.1 (9.7–10.6)	276	19.1 (17.6–20.7)
Father ever beat her mother				
No	10846	60.7 (60.0–61.4)	1325	12.5 (11.3–13.8)
Yes	6271	35.3 (34.6–36.0)	1064	17.3 (15.8–18.7)
Do not know	648	4.0 (3.7–4.3)	60	12.5 (11.2–13.7)
**Community level**				
Place of residence				
Urban	3741	25.9 (25.2–26.5)	604	17.5 (16.0–19.0)
Rural	14024	74.2 (73.5–74.8)	1845	13.1 (11.8–14.4)

% calculated based on the weighted samples; % for women who sought help calculated for respective category; numbers are rounded off to one decimal place

### Predictors of help-seeking by women facing IPV

The study used Poisson regression analysis to investigate the relationship of different socio-demographic variables with help-seeking (Table 2).

**Table 2:** Predictors of help-seeking among women facing IPV

Variables	Unadjusted PR		Adjusted PR	
	***β* (95% CI)**	***p*-value**	***β* (95% CI)**	***p*-value**
**Individual level**				
** Marital status of woman**				
Currently married	*Reference*			
Formerly married	2.0 (1.6–2.2)	< 0.001	Not applicable	
** Woman currently working**				
No	*Reference*			
Yes	1.5 (1.4–1.6)	< 0.001	1.1 (0.9–1.3)	0.274
**Relationship-household level**				
** Number of living children**				
0	*Reference*			
≤ 2	0.8 (0.7–0.9)	0.001	0.8 (0.6–1.1)	0.188
> 2	0.7 (0.6–0.9)	< 0.001	0.8 (0.6–1.1)	0.165
** Husband drinks alcohol**				
No	*Reference*			
Yes	1.9 (1.8–2.1)	< 0.001	1.7 (1.5–2.0)	< 0.001
** Women empowerment (makes decisions about own health, large purchases, mobility)**				
No	*Reference*			
Yes	0.8 (0.8–0.9)	< 0.001	0.9 (0.8–1.0)	0.082
** Wealth quintile**				
Poorest	*Reference*			
Poorer	1.1 (1.0–1.2)	0.317	1.1 (0.9–1.3)	0.477
Middle	1.1 (1.0–1.3)	0.065	1.1 (0.9–1.3)	0.443
Richer	1.1 (1.0–1.3)	0.073	1.2 (1.0–1.5)	0.079
Richest	1.4 (1.2–1.6)	< 0.001	1.4 (1.1–1.9)	0.009
** Father ever beat her mother (parental IPV)**				
No	*Reference*			
Yes	1.4 (1.3–1.5)	< 0.001	1.3 (1.1–1.4)	< 0.001
Do not know	0.8 (0.6–1.0)	0.036	0.8 (0.5–1.3)	0.388
**Community level**				
** Place of residence**				
Urban	*Reference*			
Rural	0.8 (0.7–0.9)	< 0.001	0.9 (0.8–1.1)	0.266

The following covariates were adjusted in multivariate model including place of residence, respondent’s current working status, respondent’s occupation, current marital status, respondent’s age at first marriage, number of children, husband/partner worked in last 12 months, wealth index, husband consumes alcohol, respondent’s control over decision-making, and respondent witnessed IPV as a child

At the individual level and in the unadjusted model, women who were formerly married (currently widowed, separated or divorced) and were currently working were more likely to seek help. However, neither variable remained significant after adjusting for other covariates. At the relationship-household level and in the unadjusted model, women with more than two children and those who were empowered were less likely to seek help. Those who were from the richest wealth quintile were more likely to seek help. However, all three variables ceased to be significant after adjusting for other covariates. Women whose husbands drank alcohol, and those who witnessed parental IPV were more likely to seek help in both the unadjusted and adjusted models. At the community level and in the unadjusted model, women residing in rural areas were less likely to seek help, but this was not significant in the adjusted model. Table 2 shows the unadjusted and adjusted values per level. For the full table, see Additional file 2: Table S2.

### Type of IPV and source of help

The proportions of women who sought help for any form of violence (14.9%) and specifically for physical violence (14.4%) were in a similar range, while a higher proportion of women sought help for sexual violence (22.5%). Notably, only one in ten women approached formal help sources. Among those who did seek formal help, the police emerged as the primary source of help (6.94 to 8.43%), followed by doctors (1.84 to 2.71%). The majority (95%) of women who sought help opted for informal sources, among whom, three in five women sought help from their own families and three in ten from their in-laws or marital families. Table 3 presents the type of IPV with the corresponding source of help. For the full table, see Additional file 3: Table S3.

**Table 3:** Type of IPV and corresponding source of help

Description	Any violence (*N* = 17,765)		Physical violence (*N* = 17,234)		Sexual violence (*N* = 3520)	
	***n***	**% (95% CI)**	***n***	**% (95% CI)**	***n***	**% (95% CI)**
Sought any help	2449	14.2 (13.7–14.7)	2404	14.4 (13.9–14.9)	811	22.5 (21.9–23.1)
Formal help sources						
Any formal help	226	11.4 (11.0–11.9)	226	11.6 (11.1–12.1)	109	13.1 (12.6–13.5)
Police	141	6.9 (6.6–7.3)	141	7.0 (6.7–7.4)	70	8.4 (8.0–8.8)
Doctor	38	2.7 (2.4–2.9)	38	2.7 (2.5–3.0)	17	1.9 (1.7–2.1)
Informal help sources						
Any informal help	2358	95.8 (95.5–96.1)	2313	95.8 (95.5–96.0)	763	95.3 (95.0–95.7)
Own family	1496	61.3 (60.6–62.0)	1476	61.6 (60.9–62.3)	467	58.6 (57.9–59.3)
Marital family	763	30.5 (29.9–31.2)	747	30.5 (29.9–31.2)	249	29.0 (28.4–29.7)

Women can report more than one source from which they sought help; % are based on the weighted sample; numbers are rounded off to one decimal place

## Discussion

This study identified individual, relationship, household and community-level factors associated with help-seeking by women facing IPV. It also studied the association of the type of IPV with the source of help. Slightly more than 14% of all women who faced physical and/or sexual IPV sought help, implying that a much larger percentage of women faced IPV but did not seek help. The literature indicates that factors such as non-recognition of abuse, conviction that the abuse shall end on its own, anticipated stigmatization by potential sources of help and lack of alternative financial and social support sources negatively impact women’s decision to seek help [4, 16]. In a study from Goa, India [11], close to seven in ten women said they did not seek help for IPV because they did not want to cause distress to their own families. Further, the present study found that within formal sources of help, the police were the most preferred choice; it also found that more women facing sexual IPV sought help from the police compared to women facing physical IPV.

The study found the husband’s consumption of alcohol to be associated with increased help-seeking by the woman. Alcohol use is in fact a risk factor for the prevalence of IPV and also a trigger for help-seeking [41, 42]. A recent analysis [43] that identified 15 risk factors for IPV found husband’s alcohol consumption and witnessing parental IPV by the woman as two of the most significant factors associated with increased help-seeking: both these factors are reconfirmed by this present study [11]. A study [41] of factors facilitating help-seeking behaviour found that when an abusive husband consumed alcohol often, the woman was more likely to sustain eye injuries, sprains, dislocations or burns. Such severe injuries would require the woman to seek medical treatment. It is likely that she would disclose the violence to the healthcare provider and seek help, especially if providers are responsive and empathetic. Future studies could enquire if the nature and burden of physical injuries have any association with sources of formal help accessed.

This study also found that women who witnessed parental IPV were more likely to seek help for their own IPV. This is evidenced in other studies [15]: a study from Nigeria [44] found that women who witnessed parental IPV had up to 33% higher odds of help-seeking when they faced IPV themselves. They are more likely—compared to women who did not witness parental IPV—to realize that IPV usually escalates with time; this facilitates their movement through the first and second stages of help-seeking sooner. It is possible that a woman who knows her mother had faced IPV might find it easier to disclose her experience to her mother to seek help or suggestions of mitigating the abuse [44]. Future research could consider studying if women who witnessed parental IPV move more quickly through the first and second stages of help-seeking.

The study also found that women who were empowered—i.e. who made their own decisions about healthcare, purchases and mobility—were less likely to seek help than women who were not empowered. Other studies [11, 45] had similar results, viz. women who are empowered have lower odds of help-seeking. This is likely because such women are aware of their empowerment and feel that they need to take care of the social respectability of their families [2]. Empowered women could also be more likely to blame themselves for their abuse, more so if they had chosen their husbands or partners themselves [46]. Empowered women who face IPV are found to have significant chances of explaining to themselves that since they are empowered, they need to be stronger and more capable of handling their problems on their own [47, 48]. It thus seems that empowered women are doubly victimized, first by their abusive husbands or partners and then by social norms that need them to project themselves as strong, self-reliant women.

An interesting finding of this study is that more than one in ten women sought formal help. This is a significant increase over findings from earlier rounds of the NFHS. In both previous rounds of the survey (2005–2006 and 2015–2016), between 1 and 2% of women facing IPV sought formal help [10, 41]. The present study found that help-seeking specifically from the police was significantly high at almost 7%. The third round of the NFHS (2005–2006) found [41] that less than 1% of women sought help from police, while the fourth round (2015–2016) found [10] that only 0.6% of women sought help from police. The significant increase in women seeking police help in this present round merits reflection.

Various state governments in India have taken initiatives to strengthen the gender responsiveness of their police staff. In 2016–2017, the Indian Police, in collaboration with Sheffield Hallam University’s Helena Kennedy Centre for International Justice, launched a programme—‘Justice for her’—in some states in North India [49]. This training programme aims to strengthen the capacities of the police force to understand gender and build skills for adopting a gender-responsive approach towards women and children. Another initiative by the Tata Institute of Social Sciences (TISS) Special Cells in certain states such as Maharashtra and Bihar works closely with the police to transform ideas about gender and violence. Under this initiative, trained social workers are placed within police stations to support the police to deal empathetically with women and girls reporting violence. More than 100 such cells are functional in Maharashtra while each district of Bihar has, or is preparing for, such cells [50]. India also has Women’s Police Stations in many states. A review of all Women’s Police Stations in India [51] found close to 30% increase in the reporting of violence against women cases to the police: as the report said, this increase was ‘driven by an increase in reporting of domestic violence [IPV] cases’.

Further, in 2017, the Ministry of Women and Child Development, Government of India, prioritized One Stop Centres (OSC) to support women who have faced or are facing ‘physical, sexual, emotional, psychological and economic abuse’ within the family or in public places [52]. The first One Stop Crisis Centre was set up by the Center for Enquiry into Health and Allied Themes (CEHAT) at the KB Bhabha Hospital, Mumbai. Named Dilaasa—or ‘reassurance’—this model lent itself to the imagining of a one-stop safe space for survivors of violence across India [53]. This model has been scaled across other states [53], thus moving towards the creation of spaces where survivors of IPV and other forms of violence are supported, and systems are mobilized under one roof. The increase in number of women seeking support from the police instils confidence about the positive outcomes of these initiatives that the state has been prioritizing. In order to quantify the positive impact of such initiatives, future studies could assess the rates of police help-seeking especially for states where the training programmes or initiatives are actively running versus where they are yet to take off.

These findings help emphasize the need for similar initiatives to be implemented for healthcare personnel as well since women also seek help from them. Such initiatives could include equipping healthcare personnel to provide psychosocial first aid to survivors who seek help for IPV, link survivors with social workers and other relevant organizations, provide them with helpline numbers and make need-based intersectoral referrals. Interventions in India, such as Dilaasa in Maharashtra [54], Bhoomika in Kerala [55] and Sajha in Bihar [56], are focused on building the capacities of healthcare personnel to identify and support survivors of IPV.

The present study found that among informal sources, the natal family was overwhelmingly preferred by women facing IPV, and this is in sync with existing evidence [10, 11]. According to a recent systematic review of patterns of informal supporters of IPV, an estimated 75% of IPV survivors made initial disclosures to informal networks. Strong informal support systems reduce the likelihood of re-abuse. Empowering families—with information about the availability of IPV support and capacity building to provide immediate psychological support to someone disclosing IPV and seeking help—is expected to translate to a strengthened informal network that would eventually be able to provide confidence to survivors to reach out for help sooner.

Most of the limitations of this study stem from the methodology of the dataset. The data is cross-sectional and thus causal associations cannot be drawn. Second, in this round of the survey, married women below the age of 18 years were excluded from the sample. Given that in India a significant proportion of girls under the age of 18 get married, this dataset did not include help-seeking information for this sub-population. Third, as only one woman per household was interviewed, other potential survivors of violence in the same household could not be documented. Fourth, the methodology used to calculate this percentage is based on reported cases, while the actual number could be higher. Finally, the data combines responses of women who sought help only once with those who sought help multiple times.

## Conclusions

Based on this analysis of data from India, it can be concluded that women whose husbands consumed alcohol and those who witnessed parental IPV were most likely to seek help. Most women preferred to seek help from the natal family. A significant finding is that among formal sources most women sought help from the police. India has been focusing on integrating gender responsiveness in police trainings, and these findings emphasize the importance of continuing prioritization of such capacity-building interventions. Finally, since families are the first port of help-seeking for most women, it would be important to continue investing in building the capacities of communities to respond to women disclosing IPV and seeking help.

## Supplementary Information

**Additional File 1:** **Table S1.** Demographic characteristics of all women who faced IPV (*N*=17,765) and prevalence of help-seeking.

**Additional File 2: Table S2.** Predictors of help-seeking among women facing IPV.

**Additional File 3:** **Table S3.** Type of IPV and source of help.

## Data Availability

The analysis is based on the freely available data on the DHS Program site (https://dhsprogram.com/data/dataset/India_Standard-DHS_2020.cfm?flag=0).

## Abbreviations

CI: Confidence interval
IPV: Intimate partner violence
NFHS: National Family Health Survey
OR: Odds ratio
PR: Prevalence ratio
SD: Standard deviation
WHO: World Health Organization
