# Supplementary material for: Factors associated with help-seeking by women facing intimate partner violence in India: findings from National Family Health Survey-5 (2019–2021)
Source: BMC Glob Public Health. 2024 Apr 17;2:25. doi: 10.1186/s44263-024-00056-3 (PMC11622889; doi:10.1186/s44263-024-00056-3)
Supplement: Supplementary file 1 — Additional File 1: Table S1. Demographic characteristics of all women who faced IPV (N=17,765) and prevalence of help-seeking. [file 44263_2024_56_MOESM1_ESM.docx]

**Factors associated with help-seeking by women facing Intimate Partner Violence in India:
findings from National Family Health Survey-5**

**Additional File 1**

**Table S1: Demographic characteristics of all women who faced IPV (N=17,765) and prevalence of help-seeking**

| **Description** | **All women who faced physical and/or sexual violence** | | **Women who sought help** | |
| --- | --- | --- | --- | --- |
|  | **n** | **% (95% CI)** | **n** | **% (95% CI)** |
| Overall | 17765 |  | 2449 | 14.2 |
| **Individual level** | | | | |
| Age | | | | |
| <=19 Years | 202 | 1.7 (1.5 - 1.9) | 25 | 15.2 (13.8 - 16.5) |
| 20-29 Years | 5102 | 27.6 (27.0 - 28.3) | 660 | 13.4 (12.1 - 14.7) |
| 30-39 Years | 7268 | 38.4 (37.7 - 39.1) | 1016 | 14.4 (13.1 - 15.8) |
| 40-49 Years | 5193 | 32.3 (31.6 - 33.0) | 748 | 14.6 (13.2 - 15.9) |
| Marital status of women | | | | |
| Currently married | 16502 | 91 (90.6 - 91.4) | 2129 | 13.1 (11.8 - 14.4) |
| Formerly married | 1263 | 9 (8.6 - 9.4) | 320 | 25.2 (23.6 - 26.9) |
| Highest educational level of women | | | | |
| No education | 6671 | 37.3 (36.6 - 38.0) | 904 | 13.6 (12.3 - 14.9) |
| Primary | 2911 | 15.8 (15.2 - 16.3) | 411 | 13.6 (12.2 - 14.9) |
| Secondary | 7149 | 41.4 (40.6 - 42.1) | 994 | 14.7 (13.3 - 16.0) |
| Higher | 1034 | 5.6 (5.3 - 6.0) | 140 | 16.7 (15.3 - 18.2) |
| Currently working | | | | |
| No | 11272 | 63.5 (62.8 - 64.2) | 1311 | 11.5 (10.3 - 12.7) |
| Yes | 6493 | 36.5 (35.8 - 37.2) | 1138 | 18.9 (17.4 - 20.4) |
| **Relationship-household level** | | | | |
| Husband education level | | | | |
| No education | 4282 | 26.3 (25.6 - 26.9) | 624 | 14.3 (13.0 - 15.7) |
| Primary | 3079 | 17.9 (17.4 - 18.5) | 425 | 14.3 (12.9 - 15.6) |
| Secondary | 8819 | 46.9 (46.2 - 47.7) | 1208 | 14.5 (13.1 - 15.9) |
| Higher | 1512 | 8.5 (8.1 - 8.9) | 178 | 12.3 (11.1 - 13.6) |
| Don't know | 73 | 0.4 (0.3 - 0.5) | 14 | 9.3 (8.2 - 10.5) |
| Earns more than husband | | | | |
| More than him | 1131 | 6.4 (6.0 - 6.7) | 215 | 23.2 (21.6 - 24.8) |
| Less than him | 3260 | 17.9 (17.4 - 18.5) | 553 | 17.1 (15.6 - 18.5) |
| About the same | 973 | 5.5 (5.1 - 5.8) | 123 | 14.3 (13.0 - 15.7) |
| Husband doesn't bring in money | 322 | 1.7 (1.5 - 1.9) | 61 | 16.1 (14.7 - 17.5) |
| Don't know | 66 | 0.3 (0.2 - 0.4) | 10 | 39.3 (37.5 - 41.2) |
| Number of living children | | | | |
| 0 | 1063 | 6.1 (5.7 - 6.4) | 190 | 18.1 (16.6 - 19.6) |
| <=2 | 9326 | 52.1 (51.4 - 52.8) | 1279 | 15.2 (13.8 - 16.6) |
| >2 | 7376 | 41.8 (41.1 - 42.5) | 980 | 12.4 (11.1 - 13.7) |
| Owns a house alone or jointly | | | | |
| Does not own | 8332 | 50.2 (49.5 - 51.0) | 1164 | 14.5 (13.1 - 15.9) |
| Alone only | 3087 | 17.8 (17.2 - 18.3) | 480 | 15.8 (14.4 - 17.2) |
| Jointly only | 3156 | 15.9 (15.3 - 16.4) | 430 | 13.0 (11.7 - 14.3) |
| Both alone and jointly | 3190 | 16.1 (15.6 - 16.6) | 375 | 12.6 (11.4 - 13.9) |
| Owns land alone or jointly | | | | |
| Does not own | 10521 | 62.4 (61.7 - 63.2) | 1501 | 14.5 (13.1 - 15.9) |
| Alone only | 2032 | 12.0 (11.5 - 12.4) | 286 | 14.3 (13.0 - 15.7) |
| Jointly only | 2567 | 12.4 (11.9 - 12.8) | 345 | 13.1 (11.8 - 14.4) |
| Both alone and jointly | 2645 | 13.2 (12.8 - 13.7) | 317 | 13.7 (12.3 - 15.0) |
| Husband drinks alcohol | | | | |
| No | 9838 | 58.8 (58.0 - 59.5) | 959 | 10.2 (9.0 - 11.4) |
| Yes | 7927 | 41.3 (40.5 - 42.0) | 1490 | 19.9 (18.4 - 21.5) |
| Women empowerment (decision about own health, large purchase, visit to family) | | | | |
| No | 5619 | 32.1 (31.4 - 32.7) | 812 | 14.1 (12.8 - 15.5) |
| Yes | 10883 | 58.9 (58.2 - 59.6) | 1317 | 12.6 (11.3 - 13.8) |
| Women justify wife beating | | | | |
| No | 16605 | 92.8 (92.5 - 93.2) | 2286 | 14.3 (13.0 - 15.7) |
| Yes | 1160 | 7.2 (6.8 - 7.6) | 163 | 12.5 (11.2 - 13.8) |
| Wealth quintile | | | | |
| Poorest | 5029 | 25.5 (24.9 - 26.2) | 636 | 13.5 (12.1 - 14.8) |
| Poorer | 4565 | 25.6 (25.0 - 26.2) | 611 | 12.3 (11.0 - 13.6) |
| Middle | 3723 | 21.7 (21.1 - 22.2) | 525 | 15.2 (13.8 - 16.5) |
| Richer | 2828 | 17.1 (16.6 - 17.7) | 401 | 14.0 (12.7 - 15.4) |
| Richest | 1620 | 10.1 (9.7 - 10.6) | 276 | 19.1 (17.6 - 20.7) |
| Father ever beat her mother | | | | |
| No | 10846 | 60.7 (60.0 - 61.4) | 1325 | 12.5 (11.3 - 13.8) |
| Yes | 6271 | 35.3 (34.6 - 36.0) | 1064 | 17.3 (15.8 - 18.7) |
| Don't know | 648 | 4.0 (3.7 - 4.3) | 60 | 12.5 (11.2 - 13.7) |
| **Community level** | | | | |
| Place of residence | | | | |
| Urban | 3741 | 25.9 (25.2 - 26.5) | 604 | 17.5 (16.0 - 19.0) |
| Rural | 14024 | 74.2 (73.5 - 74.8) | 1845 | 13.1 (11.8 - 14.4) |

*Note: % calculated based on the weighted samples; % for women who sought help calculated for respective category; numbers are rounded off to one decimal place*
