# Supplementary material for: Factors associated with help-seeking by women facing intimate partner violence in India: findings from National Family Health Survey-5 (2019–2021)
Source: BMC Glob Public Health. 2024 Apr 17;2:25. doi: 10.1186/s44263-024-00056-3 (PMC11622889; doi:10.1186/s44263-024-00056-3)
Supplement: Supplementary file 2 — Additional File 2: Table S2. Predictors of help-seeking among women facing IPV. [file 44263_2024_56_MOESM2_ESM.docx]

**Factors associated with help-seeking by women facing Intimate Partner Violence in India:
findings from National Family Health Survey-5**

**Additional File 2**

**Table S2: Predictors of help-seeking among women facing IPV**

| **Variables** | | | **Unadjusted PR** | | | | **Adjusted PR** | |
| --- | --- | --- | --- | --- | --- | --- | --- | --- |
|  |  |  | **β (95% CI)** | | **p-value** | | **β (95% CI)** | **p-value** |
| **Individual level** | | | | | | | | |
| **Marital status of woman** | | | |  | | | | |
|  | | Currently married | *Reference* | | | | | |
|  | | Formerly married | 1.96 (1.75 - 2.21) | | <0.001 | Not applicable | |  |
| **Woman currently working** | | | | | | | | |
|  | | No | *Reference* | | | | | |
|  | | Yes | 1.51 (1.39 - 1.63) | | <0.001 | 1.10 (0.92 - 1.32) | | 0.274 |
| **Relationship-household level** | | | | | | | | |
| **Number of living children** | | | | | | | | |
|  | 0 | | *Reference* | | | | | |
|  | <= 2 | | 0.77 (0.66 - 0.89) | | 0.001 | 0.83 (0.63 - 1.10) | | 0.188 |
|  | > 2 | | 0.74 (0.64 - 0.87) | | <0.001 | 0.82 (0.62 - 1.09) | | 0.165 |
| **Husband drinks alcohol** | | | | | | | | |
|  | No | | *Reference* | | | | | |
|  | Yes | | 1.93 (1.78 - 2.10) | | <0.001 | 1.73 (1.51 - 1.98) | | <0.001 |
| **Women empowerment (makes decisions about own health, large purchases, mobility)** | | | | | | | | |
|  | No | | *Reference* | | | | | |
|  | Yes | | 0.837 (0.77 - 0.91) | | <0.001 | 0.89 (0.77 - 1.01) | | 0.082 |
| **Wealth quintile** | | | | | | | | |
|  | Poorest | | *Reference* | | | | | |
|  | Poorer | | 1.06 (0.95 - 1.18) | | 0.317 | 1.07 (0.89 - 1.27) | | 0.477 |
|  | Middle | | 1.11 (0.99 - 1.25) | | 0.065 | 1.08 (0.89 - 1.30) | | 0.443 |
|  | Richer | | 1.12 (0.99 - 1.27) | | 0.073 | 1.21 (0.98 - 1.50) | | 0.079 |
|  | Richest | | 1.35 (1.17 - 1.55) | | <0.001 | 1.446 (1.10 - 1.91) | | 0.009 |
| **Father ever beat her mother (parental IPV)** | | | | | | | | |
|  | No | | *Reference* | | | | | |
|  | Yes | | 1.39 (1.28 - 1.51) | | <0.001 | 1.27 (1.11 - 1.44) | | <0.001 |
|  | Don't know | | 0.76 (0.58 - 0.98) | | 0.036 | 0.82 (0.52 - 1.28) | | 0.388 |
| **Community level** | | | | | | | | |
| **Place of residence** | | | | | | | | |
|  | Urban | | *Reference* | | | | | |
|  | Rural | | 0.81 (0.74 - 0.90) | | <0.001 | | 0.91 (0.76 - 1.08) | 0.266 |

*Note: The following covariates were adjusted in multivariate model including place of residence, respondent’s current working status, respondent’s occupation, current marital status, respondent’s age at first marriage, number of children, husband/partner worked in last 12 months, wealth index, husband consumes alcohol, respondent’s control over decision making, respondent witnessed IPV as a child.*
