# Supplementary material for: Factors associated with help-seeking by women facing intimate partner violence in India: findings from National Family Health Survey-5 (2019–2021)
Source: BMC Glob Public Health. 2024 Apr 17;2:25. doi: 10.1186/s44263-024-00056-3 (PMC11622889; doi:10.1186/s44263-024-00056-3)
Supplement: Supplementary file 3 — Additional File 3: Table S3. Type of IPV and source of help. [file 44263_2024_56_MOESM3_ESM.docx]

**Factors associated with help-seeking by women facing Intimate Partner Violence in India:
findings from National Family Health Survey-5**

**Additional File 3**

**Table S3: Type of IPV and source of help**

| **Description** |  | | **Any violence  (N = 17765)** | | **Physical violence  (N = 17234)** | | **Sexual violence  (N = 3520)** | |
| --- | --- | --- | --- | --- | --- | --- | --- | --- |
|  |  | | **n** | **% (95% CI)** | **n** | **% (95% CI)** | **n** | **% (95% CI)** |
| Sought any help |  | | 2449 | 14.2 (13.7 - 14.7) | 2404 | 14.4 (13.9 - 14.9) | 811 | 22.5 (21.9 - 23.1) |
|  | | Formal help sources | | | | | | |
| Any formal help |  | | 226 | 11.4 (11.0 - 11.9) | 226 | 11.6 (11.1 - 12.1) | 109 | 13.1 (12.6 - 13.5) |
| Social service organization |  | | 43 | 3.3 (3.0 - 3.6) | 43 | 3.4 (3.1 - 3.6) | 15 | 2.5 (2.2 - 2.7) |
| Police |  | | 141 | 6.9 (6.6 - 7.3) | 141 | 7.0 (6.7 - 7.4) | 70 | 8.4 (8.0 - 8.8) |
| Religious leader |  | | 56 | 2.6 (2.4 - 2.8) | 56 | 2.6 (2.4 - 2.9) | 21 | 2.0 (1.8 - 2.2) |
| Lawyer |  | | 39 | 2.4 (2.1 - 2.6) | 39 | 2.4 (2.2 - 2.6) | 13 | 1.6 (1.5 - 1.8) |
| Doctor |  | | 38 | 2.7 (2.4 - 2.9) | 38 | 2.7 (2.5 - 3.0) | 17 | 1.9 (1.7 - 2.1) |
|  | | Informal help sources | | | | | | |
| Any informal help |  | | 2358 | 95.8 (95.5 - 96.1) | 2313 | 95.8 (95.5 - 96.0) | 763 | 95.3 (95.0 - 95.7) |
| Own family |  | | 1496 | 61.3 (60.6 - 62.0) | 1476 | 61.6 (60.9 - 62.3) | 467 | 58.6 (57.9 - 59.3) |
| Marital family |  | | 763 | 30.5 (29.9 - 31.2) | 747 | 30.5 (29.9 - 31.2) | 249 | 29.0 (28.4 - 29.7) |
| Current/former husband |  | | 25 | 1.2 (1.0 - 1.4) | 23 | 1.1 (1.0 - 1.3) | 9 | 1.8 (1.6 - 1.9) |
| Current/former boyfriend |  | | 4 | 0.2 (0.1 - 0.3) | 4 | 0.2 (0.1 - 0.3) | 1 | 0.2 (0.2 - 0.3) |
| Neighbour |  | | 259 | 9.1 (8.7 - 9.5) | 255 | 9.1 (8.7 - 9.6) | 99 | 9.2 (8.8 - 9.6) |
| Other |  | | 45 | 1.3 (1.1 - 1.5) | 45 | 1.3 (1.2 - 1.5) | 11 | 0.7 (0.6 - 0.8) |
| Friend |  | | 406 | 15.6 (15.1 - 16.1) | 394 | 15.5 (14.9 - 16.0) | 164 | 18.5 (18.0 - 19.1) |

*Note: Women can report more than one source from which they sought help; % are based on the weighted sample; numbers are rounded off to one decimal place*
